# Graphene-Based Sensors and Biosensors Fabricated via Pulsed Laser Deposition for Chemical and Biological Threat Detection: A Comprehensive Roadmap

**DOI:** 10.3390/s26041214

**Published:** 2026-02-13

**Authors:** Diogenes Kreusch Filho, Larissa Oliveira de Sá, Marcela Rabelo de Lima, Adriel Faddul Stelzenberger Saber, Fernando M. Araujo-Moreira

**Affiliations:** Programa de Pós-Graduação em Engenharia Nuclear, Instituto Militar de Engenharia (IME), Praça General Tibúrcio 80, Urca, Rio de Janeiro 22290-270, Brazil; kreusch.diogenes@ime.eb.br (D.K.F.); larissa.oliveira@ime.eb.br (L.O.d.S.); marcela.robelo@ime.eb.br (M.R.d.L.); faddul.adriel@ime.eb.br (A.F.S.S.)

**Keywords:** graphene, pulsed laser deposition, chemical warfare agents, biosensors, CBRN defense, nanomaterials, organophosphate nerve agents

## Abstract

**Highlights:**

**What are the main findings?**

- We propose a modular, non-linear roadmap for graphene Chemical, Biological, Radiological and Nuclear (CBRN) sensing that links planning, modeling, fabrication, multiscale diagnostics, and field validation.
- Pulsed laser deposition (PLD) is identified as a controllable, substrate-flexible route to engineer graphene thickness/defects and interfaces, improving sensor reproducibility.

**What is the implication of the main finding?**

- The roadmap provides a deployability-oriented framework to translate graphene sensing from lab demonstrations to operational CBRN platforms through iterative, feedback-driven development.
- PLD-enabled control of material properties and interfaces supports scalable fabrication strategies and more consistent device performance across substrates and batches.

**Abstract:**

Graphene-based sensors and biosensors are attractive candidates for chemical and biological threat detection due to their high surface sensitivity, rapid transduction, and low-power operation, yet real-world deployment remains constrained by cross-sensitivity, interface instability in biosensing, and limited validation under operational conditions. This review consolidates key requirements for Chemical, Biological, Radiological, and Nuclear (CBRN) detection and proposes a structured roadmap to guide the transition from laboratory demonstrations to field-relevant sensing systems. The roadmap is explicitly modular and non-linear, integrating (i) qualitative research planning and gap analysis, (ii) computational screening via molecular docking as a hypothesis-generation tool with well-defined limitations, (iii) graphene electrode fabrication and functionalization using pulsed laser deposition (PLD) to enable tunable thickness/defect engineering and strong interface control, (iv) multiscale characterization combining laboratory methods with in situ/portable diagnostics, and (v) field-oriented performance evaluation focused on response time, stability, selectivity against industrial interferents, and false-positive/false-negative behavior. Iterative feedback loops connect all modules, enabling progressive refinement of material processing, recognition chemistry, and device architecture. By framing success in terms of technology-maturity progression and operational metrics, this roadmap provides a practical, defense-relevant framework for developing deployable graphene-based CBRN sensing platforms.

## 1. Introduction

Graphene is one of the most versatile and widely studied nanocarbon materials, belonging to the same class as carbon nanotubes, fullerenes, and nanoscale graphitic structures [1]. Its exceptional electrical, chemical, and mechanical properties have enabled rapid advances in sensing technologies, particularly for applications requiring ultrahigh sensitivity and fast response times. In this context, the development of graphene-based sensors and biosensors has become increasingly relevant for the detection of hazardous chemical and biological agents associated with weapons of mass destruction (WMDs).

Beyond laboratory-driven innovation, the growing interest in advanced sensing platforms has been strongly shaped by recent global security events that exposed critical gaps in early-warning and detection capabilities for chemical and biological threats. High-profile incidents involving the confirmed use of chemical warfare agents in the Syrian conflict, which required international forensic verification, have demonstrated the consequences of delayed or insufficient detection capabilities [2,3]. In parallel, the worldwide disruption caused by the COVID-19 pandemic highlighted systemic weaknesses in biological surveillance and operational detection and response infrastructures, reinforcing the strategic importance of rapid, selective, and field-deployable detection technologies [4,5].

Within this global context, national research agendas have increasingly converged toward the development of resilient CBRN detection systems capable of operating under real-world constraints, where treaty compliance alone does not eliminate operational risk [6,7]. The scientific goals of this work align with Brazil’s National Defense Policy, which reaffirms the nation’s commitment to the elimination of chemical, biological, radiological, and nuclear weapons under international non-proliferation regimes, while preserving the right to develop and apply these technologies for peaceful purposes.

Consistent with this position, the Brazilian National Defense Strategy emphasizes the need to strengthen strategic capabilities related to national security, including counterterrorism measures and the enhancement of Chemical, Biological, Radiological, and Nuclear (CBRN) defense. Within this framework, the Brazilian Armed Forces have adopted a comprehensive CBRN defense posture encompassing prevention, early warning, detection, protection, rapid response, and crisis management.

In the Brazilian Army, CBRN responsibilities are centralized under the Terrestrial Operations Command, which oversees dedicated operational units and readiness exercises aimed at maintaining response capabilities across diverse threat scenarios.

This geographic focus is strategic: the Brazilian Southeast is the country’s most densely populated and economically critical region, home to major scientific and technological institutions and extensive chemical industrial infrastructure. Additionally, major logistical hubs, including the Port of Santos and the international airports of Galeão and Viracopos, serve as key transit points for chemical substances, further underscoring the need for advanced detection capabilities.

In comparison with large-scale, mission-oriented defense research programs in the United States, exemplified by DARPA-led and Department of Defense-funded initiatives with dedicated budget lines, multi-year timelines, and explicit transition-to-deployment objectives, and coordinated European security and defense research initiatives structured at the supranational level, the Brazilian approach to CBRN sensing research can be characterized as niche-oriented. This approach prioritizes strategic materials, fabrication routes, and application-driven innovation rather than large-volume deployment, placing particular emphasis on sensor attributes such as rapid response, high selectivity, low power consumption, and portability, which are essential for field operations under resource-constrained conditions [8,9].

The relevance of this research also extends to global arms control regimes, particularly the Chemical Weapons Convention (CWC) and Biological Weapons Convention (BWC). These treaties prohibit the development, production, stockpiling, and use of chemical and biological weapons and their precursors. However, documented violations of the CWC and persistent challenges in the verification and enforcement mechanisms of the BWC illustrate that compliance frameworks must be complemented by robust technological capabilities for independent detection and attribution [2,3,6,7]. Compliance with the CWC and BWC therefore requires member states not only to maintain strict prohibitions but also to ensure the reliable identification and destruction of any remaining chemical and biological weapon stockpiles. Achieving this objective depends on the availability of rapid, accurate, and robust detection technologies capable of operating in diverse and challenging environments.

In this context, the present work proposes a comprehensive conceptual framework for the development of graphene-based sensors and biosensors fabricated via pulsed laser deposition (PLD), emphasizing their application in the detection of chemical and biological threats. Rather than presenting a linear experimental protocol, the proposed framework synthesizes the existing literature to identify technological pathways that bridge the gap between laboratory-scale demonstrations and operational CBRN sensing systems.

Beyond its relevance to security and treaty compliance, this study contributes significantly to the academic and technological landscape. Graphene’s extraordinary physical, chemical, and electronic properties make it a disruptive platform for next-generation devices, including chemical sensors, biosensors, detectors, actuators, MEMS, and bio-NEMS [1,10,11,12]. Advancing national expertise in both graphene science and PLD-based fabrication is therefore essential. Strengthening domestic capabilities in these frontier technologies not only enhances Brazil’s CBRN readiness but also promotes innovation across multiple scientific and industrial sectors, reinforcing national sovereignty in critical areas of strategic development.

This review intentionally adopts a focused rather than exhaustive scope, prioritizing studies that address controllable fabrication, functional stability, and field relevance for CBRN sensing. Works were selected based on their contribution to deployability, reproducibility, and integration potential, rather than on publication volume alone.

## 2. Theoretical Background

In debates at the intersection of science, technology, industrial development, and defense management, the notion of technoscience is central. The term emphasizes that scientific knowledge and technological innovation are deeply entangled with political, economic, and strategic interests, particularly in the defense sector [13]. In this perspective, scientific activity is not merely a process of knowledge accumulation, but a decision-driven system in which research priorities, funding structures, and technological outputs are continuously shaped by societal demands, geopolitical constraints, and security doctrines. Scientific agendas and technological trajectories are therefore not neutral; they reflect explicit choices about which capabilities are pursued, which risks are mitigated, and which applications are prioritized.

Within Chemical, Biological, Radiological, and Nuclear (CBRN) defense, technoscience acquires special relevance because detection technologies directly mediate the transition from abstract threat awareness to actionable operational response. Key themes such as innovation management, national innovation systems, research and development (R&D), cybersecurity, and emerging dual-use technologies cannot be treated in isolation, as their interaction determines whether sensing platforms remain laboratory demonstrations or evolve into deployable systems. When chemical and biological agents can be employed as weapons, the ability to design, fabricate, and integrate advanced sensing and diagnostic technologies may determine the difference between early containment and large-scale catastrophe.

From this technoscientific perspective, the emergence of nanocarbon materials derived from graphitic carbon represents a pivotal shift in sensing research. Graphene, carbon nanotubes, fullerenes, and related structures now occupy a central position in state-of-the-art investigations of ultra-sensitive sensors and detectors for CBRN agents [14]. Their relevance extends beyond intrinsic electrical or chemical properties: nanocarbons are compatible with miniaturized, low-power, and portable architectures, which are essential requirements for field deployment under operational constraints. At the same time, their integration raises nontrivial challenges related to selectivity, stability, fabrication scalability, and system-level robustness, reinforcing the need for critical evaluation rather than purely performance-driven comparison.

Looking ahead, the convergence of nanocarbons with quantum technologies, particularly quantum sensing, illustrates how technoscientific choices can redefine detection paradigms. Quantum-enhanced sensing approaches exploit phenomena such as quantum coherence, spin-dependent interactions, and single-photon sensitivity to surpass classical detection limits, enabling access to weak signals and low-concentration agents in complex environments [15]. However, these approaches also introduce new constraints related to system complexity, environmental sensitivity, and operational maturity, which must be considered alongside potential performance gains.

In this context, technoscience provides a unifying framework to understand how national innovation capacity, geopolitical positioning, and security requirements jointly shape technological choices in CBRN sensing. Rather than offering an exhaustive tutorial on materials or devices, this section establishes the criteria by which sensing technologies are selected, combined, or excluded, such as deployability, robustness, scalability, and alignment with operational doctrine. These criteria directly inform the roadmap proposed in Section 3, which translates technoscientific constraints into concrete pathways for the development of graphene-based sensors and biosensors, including material selection, fabrication strategies, and system-level integration.

### 2.1. Chemical Weapons

Chemical weapons (CWs) comprise toxic chemicals, their precursors, and associated delivery systems intentionally designed to cause death, injury, or incapacitation through chemical toxicity rather than kinetic or explosive effects [16,17]. Unlike conventional munitions, their operational effectiveness derives from physicochemical properties such as volatility, persistence, and reactivity, which directly condition both exposure pathways and detection strategies. As a result, CWs are classified as weapons of mass destruction (WMDs) alongside nuclear, biological, and radiological threats [16,17].

From a contemporary CBRN perspective, the most critical CWs are organophosphate (OP) nerve agents and vesicant (blister) agents. OP nerve agents, including the G-series (GA, GB, GD) and the V-series (e.g., VX), are among the most toxic substances known, exhibiting mammalian toxicity far exceeding that of agricultural organophosphates [17]. G-agents are comparatively more volatile, enabling rapid vapor-phase dissemination and inhalational exposure, whereas V-agents are less volatile but significantly more persistent, leading to prolonged surface contamination and dermal exposure risks. These contrasting physicochemical behaviors impose distinct detection challenges, requiring sensors capable of both rapid vapor detection and reliable identification of low-volatility residues.

A particularly challenging subset of OP nerve agents is represented by the so-called A-series, commonly referred to as Novichok agents. These compounds were engineered to combine high potency with enhanced persistence and, critically, to evade standard detection methodologies. High-profile poisoning incidents in the last decade have demonstrated their real-world threat potential and underscored limitations in existing analytical and field-detection infrastructures [18]. From a sensing standpoint, Novichok agents exemplify a worst-case scenario: extreme toxicity at ultra-low concentrations, structural diversity that complicates spectral or chemoselective recognition, and degradation pathways that can obscure parent signatures. These features explain why conventional chemical sensors, often optimized for known G- or V-series agents, may fail to provide reliable early warning in such cases.

At the molecular level, OP nerve agents exert their primary toxic effect through inhibition of acetylcholinesterase, leading to accumulation of acetylcholine at synapses and neuromuscular junctions and triggering the characteristic cholinergic toxidrome [18,19,20]. While this biochemical mechanism underpins medical diagnosis and treatment, it is less directly exploitable for early environmental detection, as enzymatic inhibition occurs only after exposure. Consequently, sensing strategies must focus on physicochemical signatures of the agents themselves rather than downstream biological effects.

Vesicant agents, with sulfur mustard as the prototypical example, constitute a second major class of CWs [17,21]. These compounds are less volatile than many nerve agents but highly reactive, acting as alkylating agents that damage DNA, proteins, and lipids. Clinically, vesicants produce delayed-onset burns and respiratory injury, complicating timely diagnosis. From a detection perspective, their lower vapor pressure and delayed symptomatology increase the importance of sensitive surface and residue detection, as well as the ability to discriminate vesicants from benign industrial alkylating chemicals.

Beyond direct human targeting, CWs may also be employed to contaminate infrastructure, water supplies, agricultural systems, and industrial environments [16,17]. In such scenarios, detection must occur against complex chemical backgrounds containing solvents, fuels, pesticides, disinfectants, and combustion by-products. These industrial interferents represent a major source of false positives and cross-sensitivity for many conventional sensors, particularly those relying on nonspecific adsorption or bulk conductivity changes.

Taken together, these characteristics define a stringent and often conflicting set of requirements for chemical weapon detection. High volatility demands rapid sensor response and efficient sampling, whereas ultra-low lethal concentrations necessitate extremely low limits of detection. Structural diversity and persistence, especially for agents such as Novichoks and vesicants, require high selectivity and resistance to signal drift. Finally, operation in chemically noisy environments exposes the limitations of many traditional sensor technologies. These constraints motivate the exploration of advanced material platforms and sensing architectures capable of combining sensitivity, selectivity, robustness, and portability, criteria that directly inform the technological pathways discussed in the subsequent sections.

### 2.2. Biological Weapons

Biological weapons (BWs) represent one of the most complex and insidious threats to international security. Biological agents, including bacteria, viruses, and toxins, can cause widespread illness, death, and long-term social disruption. Beyond acute health impacts, their use can trigger food shortages, environmental degradation, and severe economic instability, making them a central concern in CBRN planning [5,22].

A key distinction between chemical and biological agents lies in their temporal dynamics. While most CWs exert their effects within seconds or minutes of exposure, biological agents typically exhibit incubation periods ranging from hours to days or even weeks. This latency allows for silent dissemination before clinical symptoms appear, complicating early detection and response. In addition, many biological agents replicate within the host, amplifying their impact, and can be transmitted via aerosols, contaminated water and food, or biological vectors such as insects [23].

Recognizing these dangers, the international community adopted the Biological Weapons Convention (BWC) in 1972, which prohibits the development, production, and stockpiling of biological and toxin weapons. However, the BWC faces verification and enforcement challenges, especially in light of rapid advances in biotechnology, synthetic biology, and genetic engineering, which have increased access to tools capable of modifying or synthesizing biological agents [22].

For a biological agent to be operationally viable as a weapon, it must possess certain attributes. Foremost among these is stability: the ability to withstand processing, storage, dissemination, and environmental conditions sufficiently to remain infectious or toxic. Agents must also be producible at scale, whether from natural reservoirs, commercial sources, or via advanced biotechnological processes [5]. The dual-use nature of modern biotechnology exacerbates these risks.

Modern societies are particularly vulnerable to biological threats due to high population densities, global interconnectedness, and the concentration of hazardous biological materials in research and industrial facilities. Accidental releases, as well as deliberate attacks, can overwhelm public health systems and provoke long-term socio-economic consequences [24]. The COVID-19 pandemic, though not a deliberate event, dramatically illustrated the disruptive potential of a highly transmissible pathogen and exposed weaknesses in surveillance, preparedness, and international coordination.

The U.S. Center for Disease Control and Prevention (CDC) classifies biological agents into three categories, A, B, and C, based on factors such as lethality, ease of dissemination, and availability of countermeasures. Category A agents, including Bacillus anthracis (anthrax) and variola virus (smallpox), pose the greatest risk and require Biosafety Level 4 (BSL-4) containment in many scenarios [5]. Historically, anthrax and smallpox have been central to offensive BW programs due to their lethality, environmental persistence, and potential for large-scale outbreaks [5,22,24].

Mitigating the threat of biological weapons demands a multidisciplinary approach that combines epidemiological surveillance, rapid diagnostics, medical countermeasures, robust biosecurity practices, and effective international governance. The BWC remains a cornerstone of non-proliferation efforts, but its effectiveness depends on continual adaptation to emerging scientific and technological developments [22].

Despite significant advances in biological detection technologies, the practical deployment of biosensor-based systems in real-world biological threat scenarios remains constrained by several critical limitations. Among the most relevant challenges are the stability and operational lifetime of bioreceptors, particularly when sensors are deployed outside controlled laboratory environments. Environmental variability including fluctuations in temperature and humidity, prolonged operation, and exposure to complex environmental matrices can progressively degrade biological recognition elements, compromising not only signal intensity but also measurement reliability and reproducibility over time [25]. From a practical standpoint, this limitation is frequently underestimated outside controlled laboratory validation.

These limitations become even more critical when coupled with the long incubation periods characteristic of many biological agents. During the early stages of infection, pathogen loads often remain below the detection limits of many biosensor platforms, even those demonstrating high analytical sensitivity under laboratory conditions. In practice, the probability of false-negative outcomes increases substantially during the initial phases of outbreaks, precisely when early identification is most strategically important for containment and mitigation efforts.

Recent studies in environmental and epidemiological surveillance indicate that early false-negative results are not solely attributable to instrumental limitations, but also to intrinsic biological factors, including slow replication kinetics, heterogeneous spatial distribution of targets, and temporal variability in viral or bacterial shedding [26,27]. Even in established applications such as wastewater-based monitoring using electrochemical biosensors, reliable detection typically occurs only after a significant increase in community-level biological load, revealing a critical window of vulnerability between agent introduction and effective identification, a limitation repeatedly observed during COVID-19 surveillance efforts.

In this context, early false negatives should be understood not as isolated sensor failures, but as a structural challenge inherent to early-stage biological detection. This reality underscores the necessity of multi-layered surveillance strategies that integrate biosensors with continuous environmental monitoring and complementary epidemiological approaches, thereby reducing reliance on single-point measurements and enhancing the robustness of responses to both natural and deliberate biological threats [26,27,28,29].

The COVID-19 pandemic unequivocally highlighted both the disruptive potential of highly transmissible pathogens and the limitations of conventional epidemiological surveillance systems. In this context, sensor-based approaches emerged as strategic complements to traditional clinical diagnostics. Environmental monitoring strategies, particularly wastewater-based surveillance, demonstrated a strong capacity for early detection of viral circulation at the community level. Electrochemical biosensors coupled with isothermal amplification techniques, such as loop-mediated isothermal amplification (LAMP), enabled sensitive and rapid identification of viral genetic material in environmental samples, frequently preceding the rise in clinically reported cases [26].

In parallel, population-scale surveillance studies based on sensor networks and integrated digital health platforms demonstrated the feasibility of near real-time monitoring of disease propagation dynamics. The combined use of data from distributed sensors, wearable devices, and epidemiological modeling enabled more timely and informed responses during the pandemic, reinforcing the role of sensors as strategic assets for early warning and situational awareness in both natural and deliberate biological threat scenarios [28]. Furthermore, microbial biosensors have been increasingly recognized as promising tools for diagnostics, surveillance, and epidemiology, with potential applications spanning clinical, environmental, and industrial contexts [27].

The post-COVID-19 period consolidated a paradigm shift in biosensor development, with these technologies increasingly conceived and validated as operational tools rather than purely experimental devices. Recent reviews document the maturation of electrochemical biosensors, particularly screen-printed platforms incorporating nanomaterials, as viable solutions for rapid, decentralized, and scalable biological detection [29]. These systems demonstrated robust performance in detecting viral antigens, nucleic acids, and biomarkers associated with both active infection and post-infectious syndromes, significantly expanding their relevance for public health surveillance and emergency response [30].

Within this technological landscape, graphene-based biosensors stand out due to their exceptional electrical sensitivity, high surface-to-volume ratio, and compatibility with miniaturized, low-power platforms. However, their application to biological threat detection remains challenged by issues related to long-term bioreceptor immobilization stability, signal drift at ultra-low analyte concentrations, and susceptibility to environmental interference. These challenges are further amplified in scenarios involving long incubation periods and early-stage infections, reinforcing the need to integrate graphene biosensors into multi-modal biosurveillance architectures rather than deploying them as standalone solutions [25,29,30]. These constraints highlight the gap that often exists between laboratory performance and field deployment and directly motivate the system-level, modular roadmap proposed in Section 3, which emphasizes integration, redundancy, and operational robustness over isolated sensor performance.

### 2.3. Sensors and Biosensors

The extreme toxicity and complex pathophysiology of chemical and biological warfare agents render even minimal exposure potentially catastrophic. Intentional release in warfare or terrorism, as well as accidental exposure during production, storage, or transport, can rapidly overwhelm public health and emergency response systems. Although international treaties prohibit the development and use of such agents, documented instances of stockpiling, covert deployment, and non-state actor interest underscore the persistence and evolution of the threat. In this scenario, early detection is paramount, not only to enable timely medical intervention, but also to trigger rapid containment, decontamination, and mitigation measures [31].

In this scope, sensors and biosensors play complementary but distinct roles. Chemical sensors typically rely on physicochemical interactions (adsorption, redox reactions, conductivity changes) to detect target compounds. These platforms often exhibit superior robustness under harsh environmental conditions, making them suitable for continuous or unattended field deployment [14,32,33,34]. Biosensors, in contrast, use biological recognition elements, such as enzymes, antibodies, nucleic acids, or receptors, providing high selectivity toward specific analytes [35]. While this selectivity is advantageous for discriminating structurally similar agents, it often comes at the cost of reduced operational stability [34].

**Figure 1:**
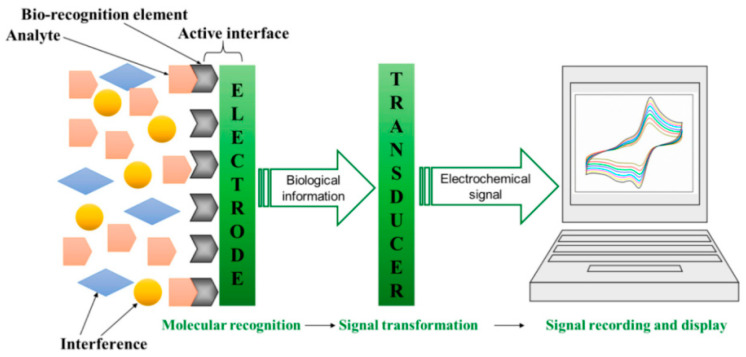
Scheme of a biosensor where the transducer works with an electrochemical signal. Image from reference [36] licensed under CC BY 4.0.

Biosensor technology is inherently interdisciplinary, combining bioactive materials with solid-state transducers, microelectronics, and data processing. Applications include clinical diagnostics, therapeutic monitoring, environmental analysis, food and beverage quality control, fermentation monitoring, and robotic sensing [14,36]. A generic biosensor consists of (i) a biorecognition layer, (ii) a transducer, and (iii) signal processing and display electronics (Figure 1). The analyte diffuses to the biorecognition element, which generates a primary biochemical response; this response is then converted into a quantifiable signal by the transducer and subsequently amplified and processed.

A major challenge common to both sensor classes in CBRN applications is the occurrence of false positives. Environmental interferents, sensor drift, cross-reactivity, and non-specific adsorption can all lead to erroneous alarms, particularly in highly sensitive systems based on nanomaterials or biological interfaces [37,38]. In real-world response scenarios, false positives may trigger unnecessary evacuations, improper allocation of limited resources, and loss of confidence in early-warning networks. Consequently, CBRN sensor development must prioritize a balanced performance, where selectivity and reliability are as critical as detection limits.

Field robustness further distinguishes CBRN sensing technologies from laboratory-based analytical devices. Sensors must operate under variable temperature, humidity, dust load, and electromagnetic interference, often with limited maintenance or recalibration. While numerous sensor platforms demonstrate excellent analytical performance under controlled conditions, their translation into field-ready systems remains limited by packaging constraints, long-term signal stability, and resistance to environmental degradation [37].

These limitations are particularly pronounced in biosensors since biological recognition elements are inherently sensitive to temperature variations, radiation, environmental contaminants, and biofouling, which may lead to denaturation or loss of activity over time. Such degradation mechanisms restrict this kind of sensor’s lifetime, posing significant challenges for long-term monitoring or deployment in austere environments. Moreover, many biosensing technologies are designed for controlled laboratory conditions and trained personnel, limiting their direct applicability in emergency response scenarios. As a result, biosensors in CBRN contexts are often more suitable for confirmatory analysis or short-term detection rather than continuous surveillance [39].

Next-generation sensors and biosensors are therefore strategic tools in CBRN defense. These devices must be capable of detecting trace levels of hazardous agents in real time, combining high sensitivity and selectivity with robustness, portability, and rapid response [14,40]. Recent advances in nanomaterials, including graphene-based platforms, have enabled the development of miniaturized sensors with enhanced sensitivity and rapid response times. However, their integration into CBRN detection systems requires careful consideration of selectivity, long-term stability, and resistance to environmental interferents. The effective deployment of such materials thus depends on design strategies that explicitly address the operational constraints of CBRN environments [33,41,42].

### 2.4. Graphene as a Nanocarbon for Sensor Applications

Graphene is a two-dimensional carbon nanomaterial composed of a single layer of sp^2^-bonded carbon atoms arranged in a honeycomb lattice. The term was introduced by Boehm and co-workers and later formalized by the International Union of Pure and Applied Chemistry (IUPAC) to refer strictly to an individual carbon layer, in contrast to graphite, which denotes a three-dimensional stack of such layers. Subsequent refinements emphasized isolated or free-standing graphene sheets, including exfoliated, transferred, suspended, or chemical vapor deposition (CVD)-grown films [1,11,12,43,44,45,46,47,48].

From an electronic standpoint, graphene is a zero-bandgap semimetal whose valence and conduction bands intersect at discrete Dirac points [12,43,49,50]. This gapless band structure underpins many of graphene’s exceptional transport properties, including ultrahigh charge-carrier mobility, but it also introduces fundamental limitations for sensing applications [51]. In particular, the absence of an intrinsic bandgap results in elevated background conductivity and increased electronic noise, which can compromise signal-to-noise ratios and hinder the discrimination of low-concentration analytes under realistic operating conditions.

Structurally, graphene can be regarded as the fundamental building block of other carbon allotropes, including carbon nanotubes, fullerenes, and graphite. Each carbon atom forms three σ-bonds through sp^2^ hybridization, while the remaining $p_{z}$ orbitals generate delocalized π- and π* bands responsible for graphene’s distinctive electronic behavior. These features contribute to its high mechanical strength, thermal conductivity, optical transparency, and chemical stability [11,12,43,52]. However, these same properties render graphene intrinsically sensitive to a broad range of adsorbed species, leading to pronounced cross-sensitivity in chemical sensing environments.

Graphene’s exceptional charge-carrier mobility—often exceeding 15,000 cm^2^·V^−1^·s^−1^ at room temperature on conventional substrates—has been widely highlighted as a key advantage for sensor applications [12,47,51,53]. Nevertheless, in practical sensing platforms, particularly those intended for field deployment, high mobility alone does not guarantee superior performance. The lack of a bandgap limits current modulation, making it difficult to suppress baseline conductivity and distinguish specific analyte-induced signals from environmental fluctuations such as humidity, temperature, or non-target chemical species.

Cross-sensitivity and limited intrinsic selectivity therefore represent major challenges for graphene-based sensors. Adsorption of chemically diverse molecules can induce similar changes in graphene’s electrical properties, increasing the likelihood of false positives in complex environments. As a result, graphene-based sensing platforms typically require additional functionalization strategies such as surface modification, selective coatings, or hybrid material architectures to enhance specificity [51].

Material selection for chemical and biological sensing in CBRN applications requires a system-level evaluation that accounts for coupled physicochemical, electronic, and operational constraints. While multiple two-dimensional material classes exhibit high intrinsic sensitivity under controlled conditions, deployable sensor performance is governed by additional factors, including electronic noise characteristics, surface chemistry stability, susceptibility to environmental interferents, processability, and technology readiness. Table 1 presents a comparative analysis of graphene, MXenes, and transition metal dichalcogenides (TMDs), highlighting how their electronic structure, surface reactivity, and fabrication maturity translate into distinct sensing behaviors and limitations. This comparison is intended to support informed material selection by explicitly mapping material properties to sensing functions and deployment requirements, rather than ranking materials based on isolated laboratory performance metrics.

From a CBRN perspective, no single two-dimensional material satisfies all operational requirements. Graphene excels in ultra-sensitive chemical detection but suffers from poor selectivity; MXenes offer superior surface chemistry and processability at the cost of environmental stability; and TMDs provide improved selectivity and electronic control, albeit with lower intrinsic sensitivity. Consequently, hybrid and multimodal sensing architectures represent the most robust pathway toward deployable CBRN sensor systems.

Despite the growing availability of alternative two-dimensional materials, the selection of graphene as the core transducer material in this work is motivated by a combination of technological maturity, operational robustness, and system-level compatibility with CBRN detection requirements. While materials such as MXenes and transition metal dichalcogenides offer attractive properties for specific sensing functions, graphene currently represents the most balanced platform for integrating high sensitivity, low power consumption, and scalable fabrication within deployable sensor architectures.

From a technological readiness perspective, graphene exhibits the highest level of material maturity among two-dimensional candidates, supported by well-established synthesis routes, including chemical vapor deposition, solution processing, and transfer techniques. This maturity enables reproducible fabrication, large-area integration, and compatibility with existing microfabrication and packaging technologies, which are essential for transitioning from laboratory prototypes to field-deployable CBRN systems. In contrast, MXenes remain limited by long-term oxidation and storage stability, while TMD-based devices often require more complex fabrication and encapsulation strategies to achieve comparable robustness.

At the operational level, graphene’s exceptionally high carrier mobility and low intrinsic electrical noise enable the rapid transduction of weak surface interactions into measurable electrical signals, a critical requirement for early warning against low-concentration chemical threats. Although graphene lacks intrinsic selectivity due to its zero bandgap, this limitation is mitigated at the system level through functionalization, hybrid architectures, and data fusion approaches, as explicitly incorporated in the proposed roadmap. Importantly, graphene’s chemical and thermal stability under a wide range of environmental conditions provides a more reliable baseline for prolonged or repeated field deployment than materials that undergo rapid surface degradation.

Furthermore, graphene offers superior flexibility in hybrid system design. Its compatibility with metallic nanoparticles, polymers, biomolecules, and complementary two-dimensional materials enables the construction of multimodal sensing platforms that combine chemical and biological detection pathways within a unified transducer framework. This adaptability is particularly relevant in CBRN contexts, where sensors must operate across chemically complex environments and respond to diverse threat classes without frequent recalibration or material replacement.

Finally, the choice of graphene is aligned with a niche-oriented innovation strategy, in which the emphasis is placed not on achieving maximum performance for a single sensing metric, but on optimizing overall system reliability, manufacturability, and integration potential. Within this framework, graphene serves not as a standalone solution, but as a robust transduction backbone around which complementary functional materials and sensing modalities can be integrated. This system-level perspective justifies its selection as the primary material platform in the present roadmap for graphene-based CBRN sensors and biosensors.

### 2.5. Pulsed Laser Deposition Technique for Graphene Production

Pulsed laser deposition (PLD) is a versatile physical vapor deposition (PVD) technique used to grow thin films by focusing high-power pulsed laser radiation onto a solid target. Following the early demonstrations of laser-induced vaporization in the 1960s, PLD gained prominence in the late 1980s when high-quality high-T_c superconducting films of YBa_2_Cu_3_O_7_ were fabricated, outperforming those produced by more established methods such as chemical vapor deposition (CVD) and molecular beam epitaxy (MBE) [68]. Since then, PLD has been widely used to grow oxides, nitrides, metals, multilayers, and complex superlattices [69].

In a typical PLD setup, a high-power pulsed laser irradiates a solid target inside a vacuum chamber, generating a plasma plume composed of atoms, ions, clusters, and droplets that expand toward a heated substrate. Deposition can occur under ultra-high vacuum or in a controlled background gas, such as oxygen, to tailor film stoichiometry. The underlying processes are governed by non-equilibrium interactions between the laser and the target: photon absorption excites electrons, which transfer energy into thermal, chemical, and mechanical channels, resulting in ablation, plasma formation, and occasionally exfoliation [44,68]. The energetic species in the plume may sputter the substrate surface and influence nucleation, growth dynamics, and defect formation [70].

**Figure 2:**
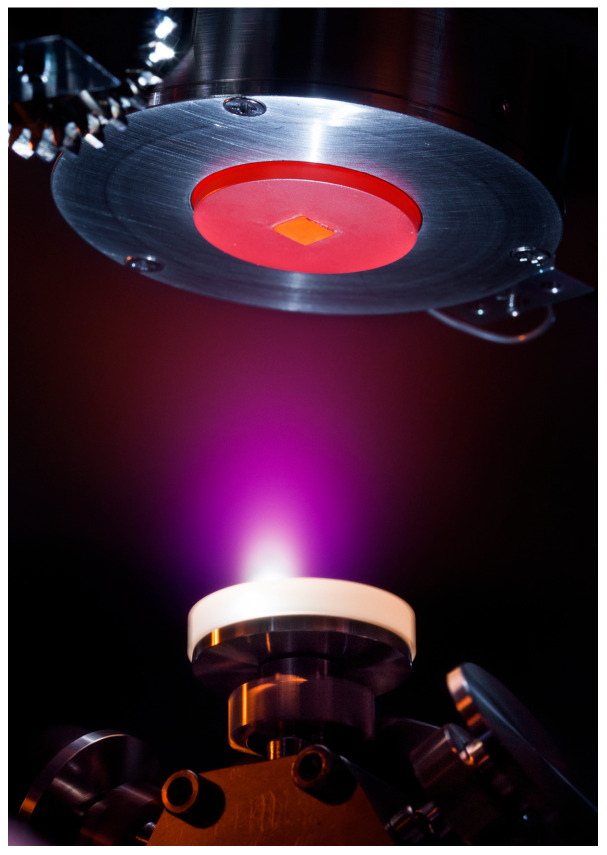
Thin oxide films are deposited with atomic-layer precision via pulsed laser deposition (PLD). In the image, a high-intensity pulsed laser targets a rotating white Al_2_O_3_ (alumina) disk, generating a plasma plume, visible as a purple cloud. This plasma expands toward a square SrTiO_3_ substrate, where the material condenses and solidifies layer by layer. The substrate, mounted on a red-hot heating stage at 650 °C, promotes the crystallinity of the growing alumina film. Image from reference [71] licensed under CC BY-SA 4.0.

**Figure 3:**
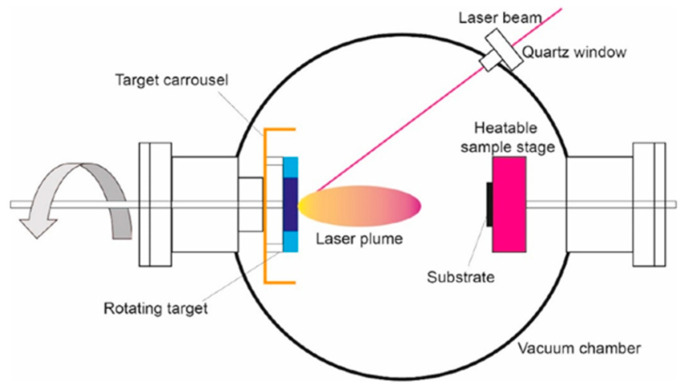
One possible configuration of a PLD chamber. Image from reference [72] licensed under CC BY 4.0.

**Figure 4:**
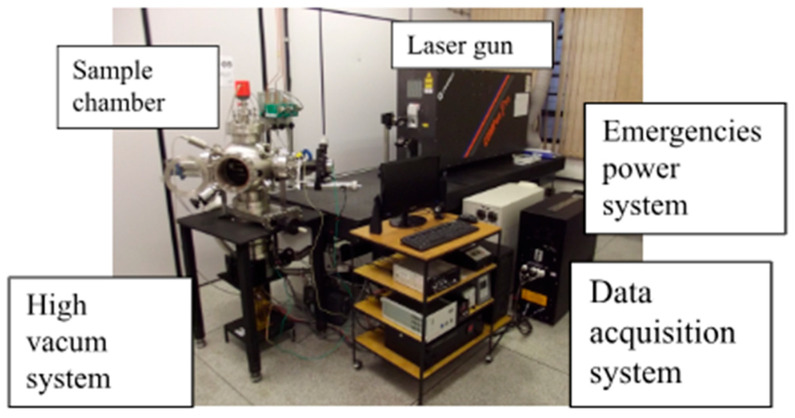
Image of a fully equipped PLD system.

Film quality depends critically on parameters such as laser fluence, repetition rate, wavelength, target composition, substrate temperature, target–substrate distance, chamber pressure, and gas composition. Chamber configurations often use rotating disk-shaped targets, though cylindrical rods with combined rotation and translation are also employed, especially for multilayer structures. A functional PLD system integrates the ablation chamber, high-vacuum infrastructure, high-power laser, substrate heating and motion control, and data acquisition (Figure 2, Figure 3 and Figure 4).

Beyond its general operational description, the distinctive character of PLD lies in its laser-driven ablation mechanism, in which the energy source is external to the deposition chamber, conferring the technique a well-defined physical identity. The laser–target interaction induces ultrafast surface heating and the ejection of a highly energetic plume composed primarily of atoms, ions, and clusters, enabling deposition under both ultra-high vacuum and controlled background gas atmospheres without compromising plasma stability [73,74]. Film growth therefore occurs intrinsically under thermodynamically non-equilibrium conditions, as the ablated species possess high kinetic energies that favor surface implantation, intermixing, and the formation of metastable phases, supersaturated solid solutions, and amorphous structures, features that define the class of materials and interfaces accessible by PLD [73,74]. As a direct consequence of this ablation dynamics, PLD exhibits high efficiency in preserving target stoichiometry in the deposited film, even for complex multicomponent systems, arising from the near-simultaneous ejection and collective transport of target constituents within the dense plasma plume, which minimizes chemical segregation [73,74]. In addition, PLD offers broad operational versatility, allowing independent control of laser parameters and deposition conditions, thereby enabling fine tuning of microstructure, interfacial properties, and growth dynamics, including layer-by-layer growth regimes that can be monitored in situ by techniques such as reflection high-energy electron diffraction (RHEED), even under relatively high background gas pressures [75].

Within the broader landscape of techniques used for carbon-based and other two-dimensional materials, and when considering graphene in particular, the choice of growth technique depends simultaneously on substrate type, film composition, uniformity requirements, thickness control, and processing conditions, and no single method is superior across all criteria [76]. In this context, PLD stands out as a highly versatile tool, particularly well-suited for the growth of chemically complex materials, for which stoichiometric transfer, energetic-species-driven growth, and access to non-equilibrium phases are desirable [74,76]. Compared to chemical vapor deposition (CVD), PLD can operate at more moderate temperatures and with shorter deposition times, favoring integration with temperature-sensitive substrates and rapid prototyping applications [74,77,78]. Relative to other PVD methods, such as thermal evaporation or sputtering, PLD offers pulsed growth in which material incorporation occurs on microsecond time scales, enabling refined control over film thickness, morphology, and composition [79].

From a cost, scale, and performance perspective, PLD can be positioned as a technique delivering high functional and structural performance, albeit with clear limitations in scalability. PVD methods aimed at continuous production, such as sputtering and evaporation, are typically associated with higher productivity and large-area industrial implementation, whereas PLD is predominantly explored in research and development environments, where fine control of local properties and interfaces is prioritized [76,79]. Although PLD relies on specialized infrastructure, it is described as a less complex and more cost-effective process when compared to higher-complexity techniques such as molecular beam epitaxy (MBE) or metal–organic chemical vapor deposition (MOCVD), while still maintaining high functional performance in the fabrication of electronically active heterostructures and interfaces [80]. Thus, although primarily applied at the laboratory scale, PLD exhibits an excellent cost-to-benefit ratio for rapid prototyping and functional device development when compared to higher-complexity techniques such as MBE or MOCVD, and within the broader comparison of routes for two-dimensional materials, representing a conscious methodological choice that prioritizes structural control, flexibility, and local performance over full-scale manufacturability, as summarized in Table 2 [79,80].

Beyond cost, scale, and performance considerations, substrate compatibility and interface control represent additional key criteria in the selection of graphene growth and deposition techniques. In this context, Table 3 provides a qualitative comparison of substrate versatility across different graphene growth, deposition, and exfoliation approaches.

In light of these comparisons, pulsed laser deposition emerges as a particularly flexible approach, motivating a closer discussion of PLD-based graphene growth strategies.

Graphene synthesis by PLD can proceed either with or without metal catalysts. In catalyst-free configurations, direct carbon deposition onto insulating substrates produces multilayer graphene at room temperature and fewer-layer films at elevated temperatures; however, precise control of layer number and uniformity remains challenging [74,83]. This intrinsic limitation is reflected in spatially heterogeneous films, as evidenced by Raman 2D mapping of graphene synthesized by PLD on silicon substrates, which reveals distinct regions of monolayer graphene, a predominance of bilayer graphene, and localized areas containing multilayer graphene. Such thickness variations are characteristic of catalyst-free PLD growth and highlight the sensitivity of the process to local growth conditions.

To address these limitations, PLD-based graphene growth can alternatively employ metal catalysts, with the choice between catalyst-free and catalyst-assisted approaches playing a decisive role in determining film morphology and overall quality [74,79]. In catalyst-assisted configurations, transition metals such as nickel or cobalt act as dissolution–precipitation media: carbon is ablated onto a metal-coated substrate, diffuses into the metal at elevated temperature, and subsequently precipitates as graphene during controlled cooling. Nickel is particularly attractive due to its high carbon solubility, lattice compatibility, thermal stability, and relatively low cost, and subsequent etching of the metal layer enables the fabrication of transfer-free graphene directly on substrates such as SiO_2_ [78,84,85]. Alternative catalysts, including cobalt, have also been explored, with optimized cooling profiles designed to match carbon diffusivity and promote few-layer graphene with reduced defect density [78].

**Figure 5:**
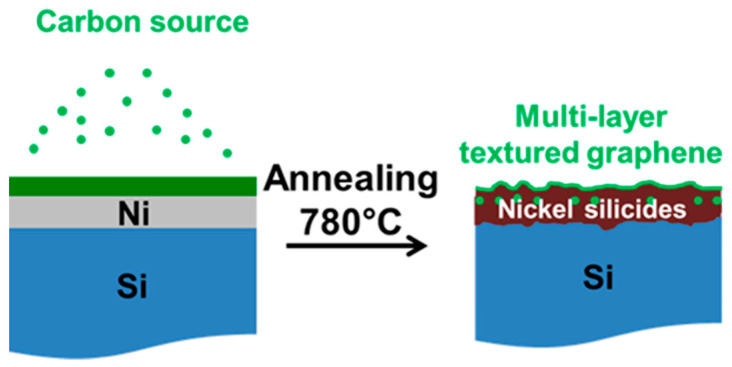
Scheme of self-organized multilayer graphene fabrication by PLD. Reproduced from reference [87] with permission from the publisher.

The number of graphene layers and film quality can be further tuned by adjusting laser fluence, with reports of monolayer graphene at ~5.66 J·cm^−2^ and bilayer at ~8.49 J·cm^−2^ have been reported [86]. More complex architectures, such as three-dimensional activated graphene electrodes, have been fabricated by sequential deposition and electrochemical treatments, illustrating the potential of PLD for advanced sensing and electrochemical applications (Figure 5) [87].

Furthermore, PLD enables in situ doping by introducing appropriate background gases (e.g., argon for p-type or nitrogen for n-type behavior), thus expanding graphene’s functional space for chemical and biosensing [88]. While catalyst-free graphene produced by PLD may exhibit variable structural quality, nickel-based catalytic approaches consistently yield films with improved uniformity and controllable layer number, reinforcing the versatility of PLD as a growth platform.

From a methodological perspective, the selection of pulsed laser deposition (PLD) for graphene synthesis in this work reflects a conscious trade-off between functional performance and scalability. As widely discussed in the literature, no graphene growth route is able to simultaneously maximize structural quality, uniformity, and large-area production, and PLD is particularly suitable for scenarios in which local control of interfaces, defects, and functional properties is critical [76,79]. For sensor applications, device performance is predominantly governed by local properties and graphene–substrate interactions rather than wafer-scale coverage, making PLD an appropriate choice due to its compatibility with diverse substrates, direct growth capability, and functional control during deposition [74]. Although PLD presents clear scalability limitations, it offers a less complex and more cost-effective implementation than higher-complexity techniques, while maintaining high local performance and an excellent cost–benefit ratio for prototyping and functional devices [80]. Accordingly, PLD is adopted in this study not as a universal solution, but as the technique best aligned with the specific objectives of developing graphene for sensor applications.

### 2.6. Graphene Sensors and Biosensors

Wearable electronics are expected to be one of the most active areas of research in the coming decade. Materials that combine high carrier mobility, optical transparency, mechanical robustness, flexibility, low weight, and environmental stability are in high demand. Graphene satisfies all these criteria and offers additional advantages, including its atomically thin geometry and the possibility of engineering diverse nanostructures (e.g., nanoribbons, porous networks) [89,90]. These attributes make graphene particularly attractive for integration into compact, low-power, and conformable sensing platforms intended for real-time monitoring in dynamic environments.

Graphene-based materials have attracted intense interest in sensors for gases, chemicals, and biomolecules, including cancer biomarkers, heavy metals, and environmental pollutants [59,60,61,62,79,80,81,82]. Owing to its high conductivity, large specific surface area, and sensitivity to surface adsorption, graphene can detect extremely small changes in its environment. In chemical sensing, the theoretical limit is the detection of single-molecule events, and micrometer-scale graphene devices have already demonstrated near-single-molecule sensitivity under controlled conditions [61,62]. However, translating such performance to operational biosensing remains challenging due to the complexity of graphene–biological interfaces.

#### Graphene–Biological Interface Challenges

When graphene is interfaced with biological recognition elements, several degradation mechanisms can limit sensor performance and reproducibility. Biological molecules immobilized on graphene surfaces are susceptible to denaturation caused by temperature fluctuations, pH variations, radiation exposure, and nonspecific interactions with the underlying carbon lattice [91,92]. In addition, weakly bound biomolecules may undergo gradual desorption, leading to signal drift and loss of sensitivity over time. Aging effects, including the oxidation of graphene derivatives and structural rearrangement of functional layers, further complicate long-term operation, particularly under continuous or repeated use [93]. These phenomena represent critical barriers for deploying graphene-based biosensors outside controlled laboratory environments.

Applications of graphene-based sensors extend beyond security and defense. In the food industry, graphene oxide (GO) and related materials have been proposed for “smart packaging” that monitors food freshness by detecting atmospheric changes associated with spoilage, potentially reducing waste and preventing foodborne illness [94,95,96]. In agriculture, graphene sensors could monitor harmful gases and environmental parameters in crop fields, helping to optimize planting strategies and manage risks [97,98]. In security and military settings, the extreme sensitivity of graphene-based sensors can be exploited to detect Chemical Warfare Agents (CWAs) and explosives at very low concentrations, enabling early warning and exposure monitoring for personnel [99,100]. Despite this broad applicability, biosensing scenarios impose stricter requirements on stability, repeatability, and resistance to biofouling than purely chemical sensing tasks.

Despite graphene’s outstanding electrical properties, its intrinsic selectivity toward specific analytes is often limited. Surface functionalization is therefore essential. Functional groups (e.g., carboxyl, amine, hydroxyl) or nanomaterials (e.g., metal nanoparticles, polymers, ionic liquids) can be introduced onto graphene to enhance recognition, binding affinity, and reduce cross-sensitivity [101,102,103]. At the same time, functionalization must be carefully engineered to preserve biomolecular activity and prevent structural degradation at the graphene–bioreceptor interface. For instance, the introduction of oxygen-containing groups has been shown to improve gas selectivity and sensitivity. Functionalization strategies also enable graphene to interface selectively with biomolecules, expanding its use to biosensing, catalysis, and advanced electronic devices [103,104,105]. As an example, Souza [104] demonstrated improved aqueous dispersion, enhanced thermal stability, and modified electrical properties of graphene using oxygenated groups, nanoparticles, and phosphonic acids.

Functionalization techniques may be broadly divided into covalent and non-covalent approaches [104,105,106]. Covalent functionalization forms new chemical bonds directly with graphene’s carbon lattice, converting some sp^2^ sites to sp^3^. This can open a bandgap and significantly alter electrical and thermal properties, as in diazotization or Diels–Alder reactions. In contrast, non-covalent strategies rely on π–π interactions, hydrophobic forces, or polymer adsorption to preserve the sp^2^ network and maintain high conductivity. Covalent methods offer stronger, more stable chemical modification at the cost of partially degrading intrinsic properties, whereas non-covalent approaches better preserve electrical performance but may provide less robust functionalization. The choice between them depends on the target application and the desired trade-offs among performance, stability, and selectivity.

To address persistent challenges related to selectivity, reproducibility, and scalability in graphene-based sensing platforms, increasing emphasis has been placed on their integration with microfluidic architectures and sensor arrays. Microfluidic systems enable precise spatiotemporal control of sample handling, including metered delivery, mixing, incubation, and isolation of sensing regions, thereby reducing reagent consumption, mitigating cross-contamination, and buffering environmental variability [107,108]. Such capabilities are particularly critical for CBRN scenarios, where analytes are often present at trace levels within complex and heterogeneous matrices.

Beyond single-sensor configurations, graphene-based sensor arrays provide a complementary strategy by enabling multiplexed and multi-analyte detection through the parallel integration of differently functionalized sensing elements within a unified platform. Array-based architectures support pattern-recognition and cross-reactivity analysis, significantly improving discrimination in chemically or biologically complex environments [107]. Recent advances in automated microfluidic sensing platforms further demonstrate that combining distributed graphene sensing elements with controlled fluidic routing and integrated data processing enhances robustness, reproducibility, and operational reliability under field-oriented constraints [109].

In the context of CBRN detection, the convergence of graphene sensors, microfluidic control, and array-based signal processing enables a transition from single-analyte, laboratory-optimized devices toward system-level sensing architectures. These architectures are better suited for applications requiring rapid screening, redundancy against false positives and false negatives, and adaptability to evolving threat profiles. Accordingly, microfluidic integration and sensor arrays should be regarded not as ancillary enhancements, but as enabling components for translating the intrinsic sensitivity of graphene into scalable, selective, and operationally viable CBRN detection systems [107,108,109].

The role of graphene in flexible gas sensors is another active research area. Graphene-based devices have been developed for detecting NO_2_, NH_3_, H_2_, H_2_S, CO_2_, SO_2_, humidity, and other gases relevant to environmental monitoring and safety [97,101,110,111,112]. In addition, graphene-based electrodes and composites are used to detect toxic heavy metal ions (e.g., Cd, Hg, Pb, Cr, Fe, Ni, Co, Cu, Ag) and volatile organic compounds (VOCs) such as nitrobenzene, toluene, acetone, formaldehyde, amines, phenols, bisphenol A (BPA), and various explosive precursors and CWAs [99,100,113,114,115,116].

One of the most critical applications of graphene-based sensors lies in security and defense. Graphene devices functionalized with polymers, metallic nanoparticles, or ionic liquids have demonstrated detection of CWAs and their simulants, such as dimethyl methylphosphonate (DMMP), an analog of nerve agents, with detection limits in the parts-per-billion (ppb) range and fast response times [117]. Their potential integration into wearable platforms makes them especially suitable for real-time monitoring of exposure in military or hazardous environments. In a related development, Dongwon Ka et al. [118] reported an e-textile sensor based on reduced graphene oxide (rGO)-coated cotton capable of detecting and distinguishing nerve agents such as GD and DMMP through changes in electrical resistance. The combination of high conductivity, mechanical flexibility, and responsiveness to chemical stimuli illustrates the promise of graphene-based materials as building blocks for next-generation CBRN sensing technologies.

Beyond conventional chemical sensing, graphene-based platforms have also demonstrated high-impact applications in emerging domains. During the COVID-19 pandemic, graphene field-effect transistor (GFET) biosensors functionalized with specific receptors achieved rapid and sensitive detection of viral proteins, illustrating their potential for real-time biomedical diagnostics [119]. This versatility extends to advanced device geometries, such as crumpled graphene FET architectures, which have been reported to achieve ultrasensitive SARS-CoV-2 detection via papain-like protease (PLpro) recognition [120]. In parallel, graphene has been explored in terahertz (THz) sensing and spectroscopy, where its tunable conductivity enables sensitive detection of biomolecular signatures and chemical agents [121]. Beyond spectroscopy-oriented sensing, single-layer graphene has also been used in electrically tunable THz metasurface absorber designs, underscoring its role in reconfigurable electromagnetic platforms that complement sensing functionalities [122]. Collectively, these examples highlight the versatility of graphene-based technologies across distinct frequency regimes and application scales.

Taken together, these developments position graphene sensors and biosensors as potential yet non-trivial components of next-generation sensing systems. Their successful deployment in CBRN and biomedical contexts depends not only on sensitivity, but also on the careful engineering of biological interfaces, long-term stability, system-level integration, and automated multi-target detection strategies.

## 3. Proposed Roadmap

**Figure 6:**
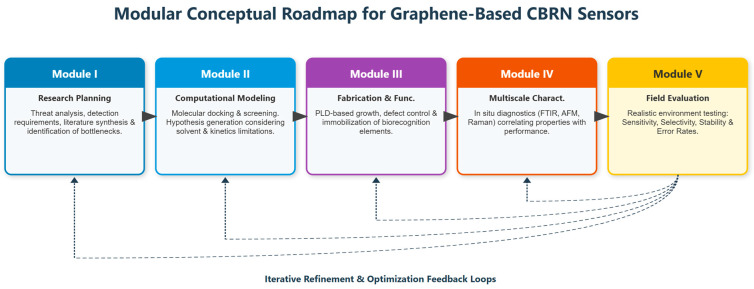
Overview of the proposed modular and non-linear roadmap for the development of graphene-based sensors and biosensors for CBRN detection.

This section introduces a structured conceptual roadmap for the development of graphene-based sensors and biosensors for chemical and biological threat detection, schematically summarized in Figure 6. As illustrated in the figure, the roadmap is conceived as a modular and non-linear framework that integrates qualitative research and critical review, computational modeling, graphene fabrication via pulsed laser deposition (PLD), multiscale characterization, and field-oriented performance evaluation. Rather than representing a fixed experimental sequence, the modules are interconnected through iterative feedback loops, reflecting an adaptive strategy in which insights gained at later stages continuously inform earlier design choices. This system-level approach is intended to guide scientific, technological, and operational progress toward robust and deployable CBRN detection platforms.

### 3.1. Module I—Research Planning

The roadmap begins with a qualitative and exploratory research module aimed at framing the technological problem space associated with chemical and biological weapons and the corresponding detection requirements within CBRN defense. Following Creswell’s principles for exploratory research [123], this module establishes the conceptual foundation that informs all subsequent technological choices.

The objectives of this module are to: (i) conduct a research on the scientific literature on chemical and biological sensors and biosensors; (ii) identify knowledge gaps and persistent technological bottlenecks; (iii) contextualize detection challenges under realistic defense and security constraints; and (iv) define research questions that guide downstream modeling, fabrication, and validation efforts. While exploratory in nature, this stage is essential for aligning material choices, sensing strategies, and performance metrics with operational needs rather than laboratory convenience.

Bibliographic analysis draws on multidisciplinary academic databases, institutional repositories, defense and strategic studies journals, and doctrinal publications from the Brazilian Armed Forces. Consultations with CBRN specialists, including personnel affiliated with the Army Command and General Staff School (ECEME), provide an additional layer of validation, ensuring that theoretical assumptions remain consistent with operational realities.

### 3.2. Module II—Computational Modeling and Its Limitations

Computational simulations constitute a second core module of the roadmap, serving as a screening and hypothesis-generation tool rather than a definitive predictor of sensor performance. Molecular docking is employed to explore potential molecular-scale interactions between chemical warfare agents (or safe simulants) and candidate biological or synthetic recognition elements, such as peptides or small proteins.

Docking simulations generate multiple ligand conformations within target binding sites and rank them using scoring functions that estimate relative affinity. In this roadmap, such simulations are used to narrow the design space and identify promising receptor candidates for experimental validation. However, it is critical to recognize their intrinsic limitations: docking typically neglects solvent dynamics, competitive adsorption, conformational flexibility under operational conditions, and time-dependent kinetics. As a result, high predicted affinity does not necessarily translate into selective or robust sensing performance in complex environments.

Accordingly, computational results are treated as guiding inputs, not design endpoints. Their primary value lies in reducing experimental search space and informing functionalization strategies, which must ultimately be validated through fabrication and empirical testing.

Importantly, docking operates at the microscopic binding scale (single ligand–receptor recognition), whereas sensor readout emerges from meso-to-macroscopic processes that include mass transport to the surface, receptor surface density and orientation, heterogeneous immobilization, nonspecific adsorption, multi-analyte competition, and device-level transduction across electrodes and arrays. Therefore, docking scores are not interpreted as direct predictors of array-level sensitivity or selectivity. Instead, docking is used to rank candidate recognition chemistries that are subsequently stress-tested under more realistic conditions through complementary multiscale steps (e.g., molecular dynamics/solvation where needed, adsorption/kinetic models for surface coverage, and empirical calibration on functionalized graphene under relevant matrices), including validation in multiplexed sensor-array formats.

### 3.3. Module III—Fabrication and Functionalization

Graphene-based electrodes are fabricated using pulsed laser deposition, as described in Section 2.6. PLD offers fine control over film thickness, defect density, morphology, and crystallinity, parameters that critically influence electrical transport, surface chemistry, and sensor reproducibility.

Key deposition variables include laser fluence, repetition rate, target composition, background gas pressure, substrate temperature, and target–substrate distance. Controlled variation in these parameters enables the production of graphene films ranging from monolayers to nanostructured multilayers with tailored properties. Prior studies demonstrating high-crystallinity SiC films suitable for epitaxial graphene growth validate the suitability of PLD for this application [124].

Post-deposition treatments, including thermal annealing and chemical enrichment of sp^2^ domains, are employed to remove adventitious adsorbates and stabilize the graphene surface. Guided by computational insights, selected biorecognition elements are immobilized using carbodiimide chemistry or Au–thiol interactions in graphene–AuNP hybrid architectures, enabling the integration of material engineering with molecular selectivity.

### 3.4. Module IV—Multiscale Characterization and In Situ Diagnostics

Characterization is performed through a multiscale strategy combining laboratory-based and portable techniques. Structural and chemical analyses include FTIR spectroscopy to confirm surface functionalization [125] and AFM to assess morphology, roughness, and defect distribution at the nanoscale [126]. Electrochemical performance is evaluated using cyclic voltammetry and differential pulse voltammetry to probe electron-transfer kinetics, sensitivity, and selectivity [127,128].

To bridge laboratory characterization and field deployment, the roadmap explicitly incorporates in situ and portable diagnostic tools, such as handheld Raman spectroscopy and portable electrochemical analyzers. Raman spectroscopy is particularly valuable for monitoring graphene quality, defect evolution, and surface chemistry under operational conditions, enabling real-time assessment of sensor stability and degradation during deployment.

### 3.5. Module V—Field-Oriented Performance Evaluation

Operational validation constitutes the final module of the roadmap, focusing on performance under realistic environmental conditions rather than idealized laboratory settings. Portable sensing units incorporating graphene-based electrodes, compact analyzers, and wireless telemetry are evaluated in urban, industrial, and rural environments representative of potential CBRN scenarios.

Field trials emphasize metrics directly relevant to deployment: response time, lowest detectable concentration, selectivity against industrial interferents, signal stability, recovery behavior, and robustness under mechanical and thermal stress. Cross-validation with laboratory reference instrumentation enables quantitative assessment of accuracy and false-positive/false-negative rates. Feedback from this module informs iterative refinement across all previous modules, reinforcing the non-linear nature of the roadmap.

### 3.6. Expected Outcomes and Technology Readiness Progression

Rather than defining fixed experimental deliverables, the expected outcomes of the proposed roadmap are framed in terms of progressive technological capability development, analogous to defense-oriented technology maturity frameworks metrics commonly employed in defense and dual-use innovation programs. In this context, progress is assessed through demonstrated functionality, robustness, and operational relevance, rather than the completion of isolated experimental tasks.

At the conceptual and modeling maturity level, the expected outcome is the establishment of a validated design space for graphene-based chemical and biological sensors. Computational screening and qualitative analysis are expected to yield multiple candidate sensing architectures exhibiting predicted affinity, selectivity, and stability above literature benchmarks. At this stage, success is defined by the reduction in design uncertainty and the identification of viable sensing strategies rather than by absolute performance metrics.

At the materials and fabrication maturity level, progress is characterized by the development of reproducible PLD-based graphene growth protocols with controlled thickness, defect density, and surface chemistry. Expected outcomes include consistent film quality across multiple deposition runs and demonstrable tunability of electrical and surface properties through process parameter control. Structural and spectroscopic characterization serve as indicators of fabrication robustness and repeatability, marking a transition from proof-of-concept materials to engineering-grade sensor substrates.

At the laboratory validation maturity level, the roadmap targets the demonstration of functional sensor prototypes under controlled conditions. Expected outcomes include electrochemical response profiles exhibiting low detection limits, rapid response times, and measurable selectivity toward representative chemical warfare agent simulants or biological targets. At this stage, performance is evaluated relative to state-of-the-art laboratory benchmarks, with emphasis on signal stability, reproducibility, and resistance to common interferents rather than on absolute optimization.

Advancement to the field-relevant maturity level is defined by successful operation of portable sensor systems under realistic environmental conditions. Expected outcomes include stable sensor performance in the presence of temperature fluctuations, humidity variation, particulate matter, and volatile organic compound backgrounds. Demonstrated metrics include response time, recovery behavior, false-positive and false-negative rates, and operational stability over extended monitoring periods (on the order of tens of hours). Validation at this level signals readiness for integration into operational CBRN detection workflows.

Across all maturity levels, iterative feedback loops are a defining feature of the roadmap. Insights from laboratory and field testing are expected to inform refinements in computational models, surface functionalization strategies, and device architecture, reinforcing the non-linear nature of technological development. In this sense, the roadmap does not prescribe a single pathway to deployment but instead defines a structured space within which multiple sensing solutions can mature toward operational readiness.

Collectively, these outcomes delineate a coherent progression from conceptual design to field-validated sensing capability. By framing success in terms of demonstrated technological maturity rather than discrete experimental milestones, the proposed roadmap supports the development of robust, adaptable, and operationally relevant graphene-based sensors for chemical and biological threat detection in CBRN defense contexts. Importantly, the structure of this roadmap is directly informed by the physicochemical, biological, and operational constraints identified in Section 2, ensuring coherence between foundational analysis and technological development.

## 4. Conclusions

In this work, a comprehensive roadmap was proposed for the development of graphene-based sensors and biosensors tailored to the demanding requirements of CBRN defense. Starting from a critical examination of chemical and biological warfare agents, including recent global triggers such as the Syrian conflict and the COVID-19 pandemic, the study framed the need for rapid and ultra-sensitive detection technologies within the context of international non-proliferation regimes and Brazil’s national security priorities. Conceptual perspectives from technoscience highlighted how innovation, guided by geopolitical and economic imperatives, shapes strategic capabilities in defense. Within this landscape, graphene emerged as a robust transducer platform, evaluated systematically against alternative 2D materials like MXenes and TMDs.

The proposed framework integrates a non-linear approach comprising in silico design, precision thin-film fabrication via pulsed laser deposition (PLD), and multi-level performance assessment linked by iterative feedback loops. Molecular docking simulations serve as a screening tool to identify promising receptor–analyte pairs, while acknowledging limitations in solvent dynamics and scale. These insights inform the fabrication of graphene films, where PLD provides superior interface control and substrate versatility compared to traditional PVD methods. Structural and electrochemical characterization—using FTIR, AFM, and voltammetry—is complemented by in situ field diagnostics like handheld Raman spectroscopy to monitor sensor degradation.

In the final stage, portable detection units integrating the optimized graphene electrodes and handheld electronics must be tested in realistic conditions in collaboration with CBRN Defense operational specialists. Field trials will provide quantitative metrics on detection limits, selectivity, and robustness, while addressing critical challenges like the graphene–biological interface stability and cross-sensitivity.

Overall, this roadmap aims not only to advance the frontiers of graphene functionalization and sensor engineering, but also to strengthen Brazil’s scientific and strategic capabilities in the face of evolving CBRN threats. By coupling computational chemistry, materials science, and defense-oriented field validation within a unified framework, the proposed approach is positioned to deliver cost-effective, high-performance detection systems. Future work should prioritize interdisciplinary collaboration and technology transfer to ensure that the concepts and prototypes outlined here can be translated into deployed countermeasures, thereby reinforcing both national security and international non-proliferation efforts.

## Figures and Tables

**Table 1:** Comparative Assessment of 2D Materials for Chemical and Biological Sensing in CBRN Applications.

Criterion	Graphene/GO/rGO [10,54,55,56,57,58]	MXenes (e.g., Ti_3_C_2_T_x_) [59,60,61]	TMDs (e.g., MoS_2_, WS_2_) [62,63,64,65,66,67]
Electronic nature	Semi-metallic (zero bandgap)	Metallic/semi-metallic	Semiconducting (finite bandgap)
Intrinsic sensitivity	Extremely high (surface fully exposed)	Very high (metallic conductivity + active surface)	High (strong surface charge modulation)
Intrinsic selectivity	Very low; requires functionalization	Low–moderate; tunable via surface terminations	Moderate; bandgap improves discrimination
Surface chemistry	Chemically inert (graphene); GO/rGO tunable via oxygen groups	Rich, intrinsic terminations (–O, –OH, –F)	Moderately reactive; defect- and phase-dependent
Chemical sensing (CW/TICs)	Excellent for volatile agents; fast response	Excellent sensitivity; humidity cross-sensitivity	Good at room temperature; slower kinetics
Biological sensing (BW)	Widely demonstrated; strong electrochemical platforms	Highly promising for electrochemical biosensing	Strong for FET and electrochemical biosensors
Bioreceptor immobilization stability	Moderate; prone to drift and biofouling	Moderate; limited by MXene oxidation	Moderate–good; depends on encapsulation
Environmental stability	High (graphene); lower for GO/rGO	Limited (oxidation in air/water)	Moderate; sensitive to humidity/oxygen
Processability/scalability	Good (CVD, printing, transfer)	Excellent (solution-processable)	Moderate (transfer, exfoliation complexity)
Power consumption	Very low	Very low	Very low
Signal drift in field conditions	Significant without compensation	Significant due to aging/oxidation	Moderate; more stable than graphene
Typical failure modes	Cross-sensitivity, false positives	Oxidation, degradation over time	False negatives at early-stage detection
Technology maturity	High (materials); medium (systems)	Medium (materials and systems)	Medium (devices); low–medium (systems)
Best-suited CBRN role	Rapid chemical warning, transducer layer	Functional layer for biosensing and hybrid sensors	Selective sensing, low-power bio-detection
Overall suitability	High for CW, moderate for BW	High potential, stability-limited	High for BW, moderate for CW

**Table 2:** Qualitative comparison of 2D material growth, deposition, and exfoliation techniques. Adapted from [79] with data collected from [74,76,79,80,81].

Technique	Typical Produced Size	Production Speed	Thickness Homogeneity	Complexity (Critical Temperature & Vacuum)	Qualitative Cost (Equipment & Consumables)
Mechanical exfoliation	1–10 µm flakes	Low	Low	Low (Room temperature—RT, no vacuum)	Low
Chemical exfoliation	1–10 µm flakes (dispersed)	Moderate	Moderate	Low (RT, no vacuum)	Low–Moderate
Liquid exfoliation	1–10 µm flakes (dispersed)	Moderate	Moderate	Low (RT, no vacuum)	Low–Moderate
CVD	>10 cm continuous films	Low	Very high	High (high temperature, controlled atmosphere/vacuum)	High
PVD (thermal evaporation/sputtering)	>10 cm films	Moderate	High	Moderate (moderate temperature, high vacuum)	Moderate
PLD	Up to ~1 cm films	High	High	Moderate (moderate temperature, high vacuum)	Moderate

**Table 3:** Qualitative comparison of substrate versatility for graphene growth, deposition and exfoliation techniques [74,76,79,80,81,82].

Technique	Substrate Versatility	Typical Substrate Types	Remarks
Mechanical exfoliation	Low	SiO_2_/Si (mainly)	Transfer required; commonly used for optical contrast and layer identification; limited control over interface and placement
Chemical exfoliation	Low–Moderate	Various substrates (after deposition)	Requires post-processing and film transfer; weak interface control
Liquid exfoliation	Moderate	Polymers, glass, flexible substrates (via coating)	Substrate versatility enabled by solution processing, not direct growth
CVD	Moderate	Metals (Cu, Ni), transferred to SiO_2_, polymers	Growth typically on metals; substrate versatility achieved via transfer
PVD	Moderate–High	Metals, oxides, semiconductors	Direct deposition possible; limited graphene crystallinity
PLD	High	Oxides, semiconductors, insulators, metals	Direct growth and strong interface control; compatible with diverse substrates

## Data Availability

The original contributions presented in this study are included in the article. Further inquiries can be directed to the corresponding author.
